# An updated radiosynthesis of [^18^F]AV1451 for tau PET imaging

**DOI:** 10.1186/s41181-017-0027-7

**Published:** 2017-06-06

**Authors:** Andrew V. Mossine, Allen F. Brooks, Bradford D. Henderson, Brian G. Hockley, Kirk A. Frey, Peter J. H. Scott

**Affiliations:** Division of Nuclear Medicine, Department of Radiology, University of Michigan, Ann Arbor, MI 48109 USA

**Keywords:** Tau imaging, Brain PET, [^18^F]T807, Flortaucipir F18, Fluorine-18 radiochemistry, Automated radiosynthesis

## Abstract

**Background:**

[^18^F]AV1451 is a commonly used radiotracer for imaging tau deposits in Alzheimer’s disease (AD) and related non-AD tauopathies. Existing radiosyntheses of [^18^F]AV1451 require complex purifications to provide doses suitable for use in clinical imaging studies. To address this issue, we have modified the synthesis of [^18^F]AV1451 to use only 0.5 mg precursor, optimized the Boc-deprotection step and developed a simplified method for HPLC purification of the radiotracer.

**Results:**

An optimized [^18^F]AV1451 synthesis using a TRACERLab FX_FN_ module led to high radiochemical yield (202 ± 57 mCi per synthesis) and doses with excellent radiochemical purity (98 ± 1%) and good specific activity (2521 ± 623 Ci/mmol).

**Conclusion:**

An updated and operationally simple synthesis of [^18^F]AV1451 has been developed that is fully automated and prepares radiotracer doses suitable for use in clinical tau PET studies.

## Background

Neurodegenerative disorders are frequently associated with at least one misaggregated protein (Sweeney et al. 2017). Historically, physicians have attempted to diagnose such disorders based upon clinical symptoms, with definitive diagnosis finalized by pathologists during post-mortem examination. In recent years however, there has been considerable effort to move diagnostic strategy from clinical detection of signs and symptoms of mid-stage disease, to early detection of pharmacological biomarkers of pre-symptomatic disease (for a recent review, see: Ahmed et al. 2014). This is expected to improve patient prognosis by enabling initiation of treatment prior to loss of neuronal function (and associated cognitive decline), and can be used to support development of disease modifying therapeutics. Diagnostic tests that allow confirmation of abnormal protein deposits in pre-symptomatic disease are therefore of the utmost importance.

Tau proteins are commonly found in neurons where they promote assembly and stabilization of microtubules (for a recent review of the role of tau in physiology and pathology, see: Wang and Mandelkow 2016). However, in Alzheimer’s disease (AD) dysfunction of enzymes that are responsible for phosphorylating tau, such as glycogen synthase kinase-3, gives rise to a hyperphosphorylated version of the protein that aggregates and forms insoluble intracellular tau neurofibrillary tangles (NFTs) (Iqbal et al. 2016). Pathology studies have shown correlation between tau NFT burden and the neuronal damage/cognitive decline associated with the later stages of AD (Haroutian et al. 2007). Given this correlation, as well as the number of tau-based therapies being developed (Boutajangout et al. 2011; Panza et al. 2016), a tau PET imaging agent would allow for the selection of tau-positive AD patients for enrollment in clinical trials of such experimental treatments, and provide a means to monitor response to therapy. In addition to AD, misaggregated tau is implicated in a range of other tauopathies (e.g. progressive supranuclear palsy (PSP), corticobasal degeneration (CBD), and tau variants of frontotemporal dementia (FTD)), and a tau radiotracer can be expected to have similar impact in all of these disorders (for reviews of the role of tau in AD and non-AD tauopathies, see: Iqbal et al. 2005, 2010, 2016).

Many different tau radiotracers have been reported to date (for recent reviews, see: Ariza et al. 2015; James et al. 2015; Villemagne et al. 2015; Watanabe et al. 2015; Kolb and Andrés, 2017; Hall et al. 2017; Saint-Aubert et al. 2017). Of these, [^18^F]AV1451 (7-(6-[^18^F]fluoropyridin-3yl)-5H-pyrido[4,3-b]indole, flortaucipir F18, [^18^F]T807) developed by Siemens Molecular Imaging and Biomarker Research (Xia et al. 2013), and subsequently licensed to Eli Lilly / Avid Radiopharmaceuticals, has been among the most utilized in clinical tau PET imaging to date. It has been used to image patients with AD (see, for example: Schwarz et al. 2016), non-AD tauopathies (Marquié et al. 2017) including PSP (Passamonti et al. 2017; Smith et al. 2017) and CBD (McMillan et al. 2016), and non-tauopathies such as dementia with Lewy bodies (Gomperts et al. 2016). Methods for kinetic modeling of [^18^F]AV1451 have been developed (Shcherbinin et al. 2016; Baker et al. 2017; Barret et al. 2017; Hahn et al. 2017), and studies have also been conducted to establish biomarker endpoints (Jack et al. 2017) and human dosimetry (Choi et al. 2016). As a cautionary side note, all of this work has also identified potential off-target binding of [^18^F]AV1451 in brain areas such as the basal ganglia. Such binding may be due to specific binding of the radiotracer to monoamine oxidase A (MAO-A) (Vermeiren et al. 2015) or neuromelanin (Hansen et al. 2016), and should be accounted for during image analysis. Nevertheless, [^18^F]AV1451 PET has been used to identify distinct cortical spreading patterns of tau pathology, aiding in our understanding of how tau distribution propagates across the brain (Sepulcre et al. 2016) and to develop image-based tau staging (Cho et al. 2016; Wang et al. 2016).

In connection with an NIH-funded project developing imaging biomarkers for dementia with Lewy body patients, we had cause to make [^18^F]AV1451 available for clinical use. Reflecting its widespread use, several different syntheses of [^18^F]AV1451 suitable for clinical use have been reported. Shoup and Gao both reported syntheses of [^18^F]AV1451 from N-Boc-protected nitro precursor (Shoup et al. 2013; Gao et al. 2015), which eliminated the need for the reduction of the nitro precursor prior to purification that the original report required to facilitate purification of [^18^F]AV1451 (Xia et al. 2013). More recently, Holt et al. reported ^18^F-fluorination of N-Boc-protected trimethylammonium precursor by microwave irradiation (Holt et al. 2016). Given the commercial availability of the N-Boc-nitro precursor **1,** and its amenability to use in a standard radiosynthesis module that employs thermal heating, it was our choice for further development of a radiosynthesis of [^18^F]AV1451 **2** for routine clinical production.

While these reported synthesis of [^18^F]AV1451 represented an attractive starting point, we were unsatisfied with various aspects of these reports, including: i) ambiguity surrounding deprotection of Boc-protected [^18^F]AV1451, since both Shoup and Gao describe the Boc group being removed concomitantly with fluorination (through either thermal promotion or due to the basic reaction conditions) while Holt includes a separate acid-mediated deprotection (Shoup et al. 2013; Gao et al. 2015; Holt et al. 2016); ii) the need for solid-phase extraction (SPE) purification steps both pre- and post-semi-preparative HPLC purification, which necessitates a reconfiguration of the synthesis module in the Shoup method; iii) the Gao method used a purpose built set-up which could complicate compliance with current Good Manufacturing Practice (cGMP), and the literature report contained none of the quality control and release data required to determine if the procedure was compliant with the US Pharmacopeia; and iv) the use of an MeCN-containing mobile phase in the Gao method (our laboratory has made applying green methods when possible a priority to reduce environmental impact and risk associated with class 2 solvents such as MeCN (Stewart et al. 2015), and simplify quality control (QC) testing since eliminating such solvents reduces residual solvent analysis to an annual QC test). To address these issues, we have undertaken a detailed investigation of the radiosynthesis of [^18^F]AV1451, and herein report insights into the *N*-Boc deprotection and a new operationally simple, greener method for the radiosynthesis and purification of the radiotracer that uses a commercial synthesis module without reconfiguration, and does not utilize any class 2 solvents in the synthesis or purification.

## Methods

### Materials for synthesis and analysis

Unless otherwise stated, reagents and solvents were commercially available and used without further purification: N-Boc-protected nitro-precursor (Part No. NPPI-95-0010C) and authentic AV1451 reference standard (Part No. FPPI-95-0002A) were purchased from Huayi Isotopes/NucMedCor. Ethanol (200 proof, USP) was purchased from Decon Laboratories, Inc. Sodium chloride 0.9%, USP and sterile water for injection, USP were sourced from Hospira. Other synthesis components were obtained as follows: Sterile vials were obtained from Hollister-Stier; Millex filters were from Millipore; QMA-light and Oasis HLB 1 cc cartridges were purchased from Waters. Prior to use QMA cartridges were conditioned with ethanol (10 mL), 0.5 M NaHCO_3_ (10 mL) and sterile water (10 mL), while HLB cartridges were conditioned with ethanol (10 mL) and sterile water (10 mL).

### Radiosynthesis

**Scheme 1:**
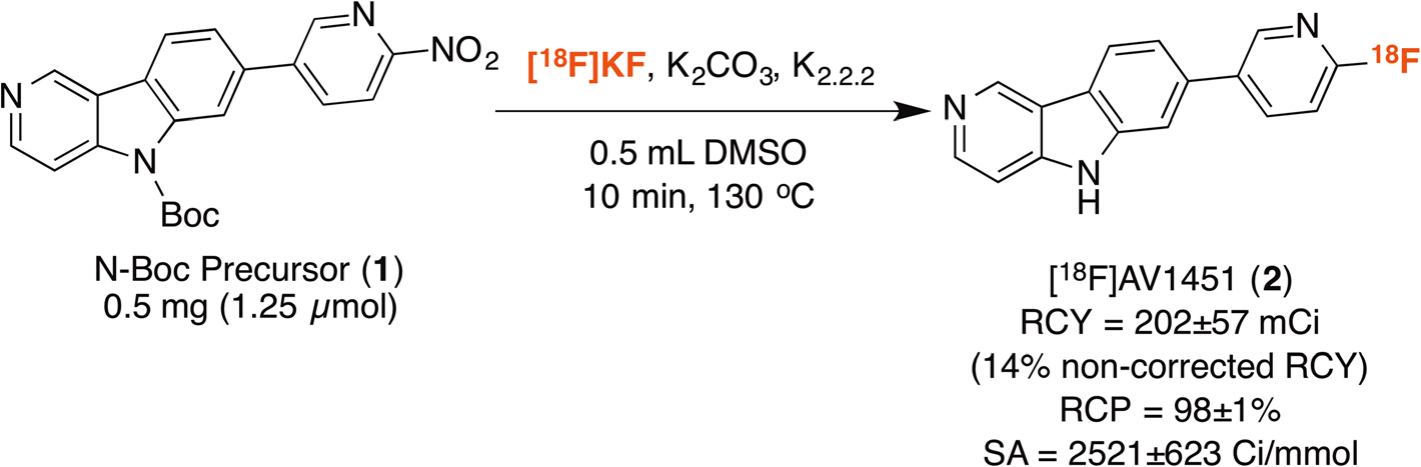
Radiosynthesis of [^18^F]AV1451 (**2**)

**Fig. 1:**
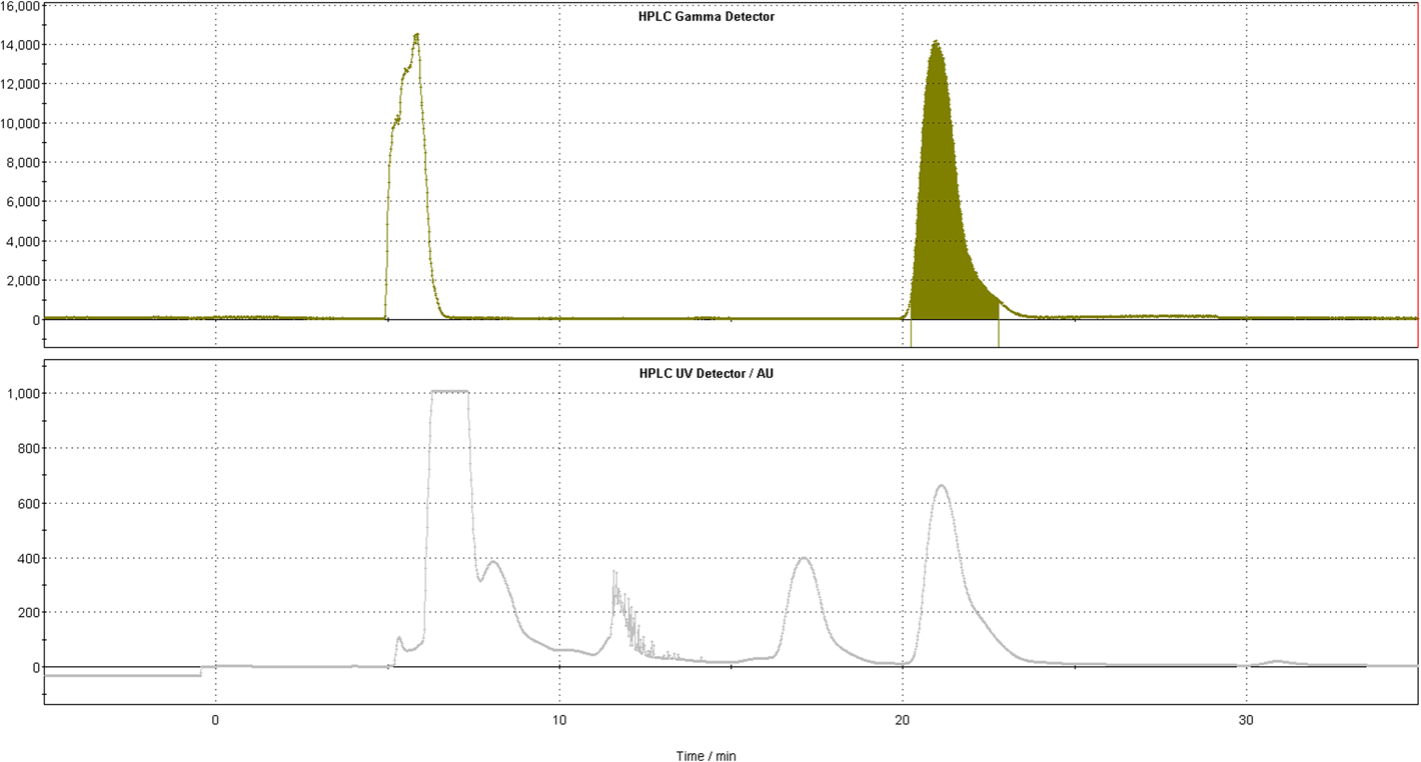
Typical semi-preparative HPLC trace from synthesis of [^18^F]AV1451

The synthesis of [^18^F]AV1451 (Scheme 1) was fully-automated using a General Electric (GE) TRACERLab FX_FN_ synthesis module loaded as follows: V1: 500 μL 7 mg/mL K_2_CO_3_ in water; V2: 1000 μL 15 mg/mL kryptofix-2.2.2 (K_2.2.2_) in ethanol; V3: 0.5 mg precursor (N-Boc nitro) in 500 μL DMSO; V6: 3000 μL semi-preparative HPLC Buffer (see below); V7: 6.5 mL buffered saline; V8: 500 μL ethanol; V9: 10 mL water; Dilution flask: 50 mL water; product vial 3 mL buffered saline. [^18^F]Fluoride (~1400 mCi) was produced via the ^18^O(p,n)^18^F nuclear reaction with a GE PETtrace cyclotron equipped with a high-yield fluorine-18 target. [^18^F]Fluoride was delivered in a 1.5-mL bolus of [^18^O]H_2_O to the synthesis module and trapped on a QMA-Light sep-pak cartridge to remove [^18^O]H_2_O. [^18^F]Fluoride was then eluted into the reaction vessel with potassium carbonate (3.5 mg in 500 μL of water). The solution of K_2.2.2_ (15 mg in 1 mL of ethanol) was added to the reaction vessel, and the [^18^F]fluoride was azeotropically dried by heating the reaction vessel to 100 °C and drawing full vacuum for 6 min. After this time, the reaction vessel was subjected to both an argon stream and a simultaneous vacuum draw for 9 min at 100 °C. The solution of AV1451 N-Boc nitro-precursor in DMSO (0.5 mg in 500 μL) was added to the dried [^18^F]fluoride, and was heated to 130 °C with stirring for 10 min. Subsequently, the reaction mixture was cooled to 50 °C, diluted with HPLC mobile phase (3 mL), and purified by semi-preparative HPLC (column: Phenomenex Gemini NX C18, 5 micron, 10x250 mm; mobile phase: 40% Ethanol 10 mM Na_2_HPO_4_ pH: 9.3 ± 0.2; flow rate: 3 mL/min; UV: 254 nm; and a typical semi-preparative trace, Fig. 1). The product peak (t_R_ = 21–22 min) was collected into the dilution flask where it was concomitantly diluted with 50 mL of sterile water. The resulting solution was passed through an Oasis HLB cartridge, which was then washed with 10 mL of sterile water. [^18^F]AV1451 was eluted with 0.5 mL of EtOH (USP for injection) and collected in the Tracerlab FX_FN_ product vial, containg 3 mL of saline (USP). The Sep-Pak was washed with 6.5 mL of saline to bring the final formulation volume to 10 mL. The final drug product was dispensed into a septum sealed, sterile, pyrogen-free glass vial through a 0.22 μm sterile filter and submitted for QC testing as outlined in the Quality Control section.

### Quality control

Quality control testing of [^18^F]AV1451 doses was conducted according to the guidelines outlined in the U.S. Pharmacopeia and as described below. Testing included visual inspection, pH, residual K_2.2.2_, chemical purity and radiochemical purity (RCP), specific activity (SA), radionuclidic identity, sterile filter integrity, bacterial endotoxin analysis, and sterility testing. Results for four process verification batches are reported in Table 1.

**Table 1:** QC Data for Process Verification Batches of [^18^F]AV1451

QC Test	Release Criteria	Batch 1	Batch 2	Batch 3	Batch 4
Visual inspection	Clear, colorless, free of particulates	Pass	Pass	Pass	Pass
pH	4.5-7.5	5.0	5.0	5.0	5.0
Radiochemical Yield (mCi)	>10 mCi	170	140	231	266
Radioactivity Conc.	>10 mCi/10 mL	34	28	46	53
AV1451 Conc.	≤5.0 μg/mL	2.5	2.0	3.5	2.5
SA (Ci/mmol)	N/A	1,789	3,228	2,788	2,278
RCP (%)	>90%	98	97	98	100
Radiochemical Identity	RRT^a^ =0.9-1.1	1.0	1.0	1.0	1.0
Radionuclidic Identity	t_1/2_ = 105-115 min	107	107	110	109
Residual K_2.2.2_	≤50 μg/mL	≤50	≤50	≤50	≤50
Filter Integrity	>44 psi	51	50	50	51
Bacterial Endotoxins	<17.5 EU/mL^b^	<2.00	<2.00	<2.00	<2.00
Sterility	Sterile	Sterile	Sterile	Sterile	Sterile

^a^RRT = Relative retention time (t_R_ [^18^F]AV1451 / t_R_ of [^19^F]AV1451 standard); ^b^ EU = Endotoxin units

### Visual inspection

Doses were visually examined and needed to be clear, colorless and free of particulate matter.

### Dose pH

The pH of the [^18^F]AV1451 doses was analyzed by applying a small amount of the dose to pH-indicator strips and determined by visual comparison to the scale provided. Dose pH was required to be between 4.5 and 7.5.

### Residual K_2.2.2_

Residual K_2.2.2_ levels in [^18^F]AV1451 doses were analyzed using the established spot test (Scott and Kilbourn 2007). Strips of plastic-backed silica gel TLC plates saturated with iodoplatinate reagent were spotted with water (negative control), 50 μg/mL K_2.2.2_ standard (positive control) and with the [^18^F]AV1451 dose. If K_2.2.2_ was present in a sample, a blue–black spot appeared. Spots for the three samples were compared and a visual determination of residual K_2.2.2_ in the dose was made; ≤50 μg/mL was acceptable.

### Chemical purity and radiochemical purity/identity

**Fig. 2:**
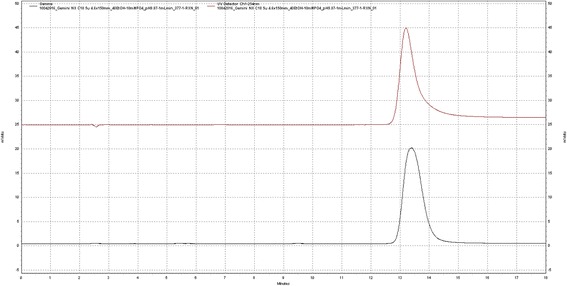
[^18^F]AV1451 QC HPLC traces (UV above, Radioactivity below) are representative of those observed

**Fig. 3:**
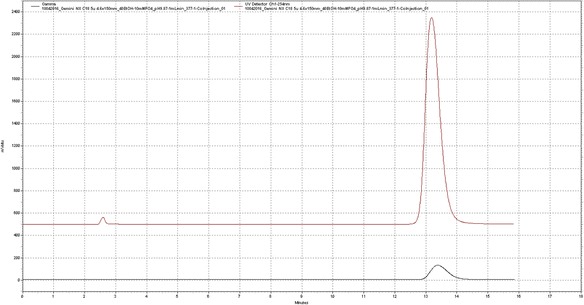
Typical Analytical HPLC trace of [^18^F]AV1451: gamma detection trace with overlay of UV trace of genuine standard (Standard UV trace above, Product Radioactivity trace below)

Chemical and radiochemical purities/identities are analyzed using a Shimadzu LC2010 HPLC equipped with a radioactivity detector and an ultraviolet (UV) detector (column: Gemini NX C18 5 micron 4.6x250 mm; mobile phase: 40% Ethanol 10 mM Na_2_HPO_4_ pH: 9.3 ± 0.2; flow rate: 1 mL/min; UV: 254 nm). A representative analytical HPLC trace is shown in Fig. 2 ([^18^F]AV1451 t_R_ = 13.2 min) and with an overlay of the UV trace of the reference standard in Fig. 3. Radiochemical purity for doses must be >90%, and identity was confirmed by comparing the retention time of the radiolabeled product with that of the corresponding unlabeled reference standard.

### Radionuclidic Identity

Radionuclidic identity was confirmed by determining the half-life of [^18^F]AV1451 doses and comparing it to the known half-life of fluorine-18 (109.77 min). Activities were measured using a Capintec dose calibrator and half-life was calculated using Eq. (1). Calculated half-life must be 105–115 min.

1 $$ {\mathrm{T}}_{1/2} = \hbox{-} \ln 2\left(\mathrm{Time}\ \mathrm{Difference}/\ \left( \ln \left(\mathrm{ending}\ \mathrm{activity}/\mathrm{starting}\ \mathrm{activity}\right)\right)\right) $$

### Sterile filter integrity (Bubble Point) test

Sterile filters from doses (with needle still attached) were connected to a nitrogen supply via a regulator. The needle is then submerged in water and the nitrogen pressure gradually increased. If the pressure was raised above the filter acceptance pressure (44 psi) without seeing a stream of bubbles, the filter was considered intact.

### Bacterial endotoxins

Endotoxin content in [^18^F]AV1451 doses was analyzed by a Charles River Laboratories EndoSafe® Portable Testing System and according to the US Pharmacopeia. Doses must contain ≤175 Endotoxin Units (EU), or ≤17.5 EU/mL.

### Sterility

Culture tubes of fluid thioglycolate media (FTM) and tryptic soy broth (TSB) were inoculated with samples of [^18^F]AV1451 and incubated (along with positive and negative controls) for 14 days. FTM was used to test for anaerobes, aerobes and microaerophiles while TSB was used to test for non-fastidious and fastidious microorganisms. Culture tubes were visually inspected on the 3rd, 7th and 14th days of the test period and compared to the positive and negative standards. Positive standards needed to show growth (turbidity) in the tubes, and [^18^F]AV1451 doses/negative controls had to show no culture growth after 14 days to be indicative of sterility.

## Results and discussion

[^18^F]AV1451 **2** features a carbazole scaffold appended with a 2-fluoropyridine moiety, which is the site of radiofluorination. The corresponding nitro-precursor **1** is an attractive starting point to synthesize the radiotracer because of its commercial availability, but is less often used than its trimethylammonium counterpart due to two major issues: i) poor solubility of the precursor in polar aprotic solvents used for S_N_Ar chemistry (displacement of -NO_2_ with -^18^F), and ii) difficulties in separation of precursor and product using semi-preparative HPLC.

Issues with solubility of the nitro-precursor have been partially resolved by N-Boc protection of the carbazole nitrogen, which improves solubility by increasing the lipophilicity of the molecule. Literature reports are unclear as to whether N-Boc protection improves radiochemical conversion or if increased yields are due to the solubility afforded (Shoup et al. 2013; Gao et al. 2015). Ambiguity also surrounds the deprotection of N-Boc-protected [^18^F]AV1451 in the literature: Shoup and Gao describe the N-Boc group being removed concomitantly with fluorination through either thermal promotion or due to the basic reaction conditions (Shoup et al. 2013; Gao et al. 2015), while Holt and co-workers add HCl following fluorination to deprotect in a separate deprotection step (Holt et al. 2016). We were also uncertain whether the acidic HPLC buffer specified in the report by Shoup might also be contributing to deprotection.

**Fig. 4:**
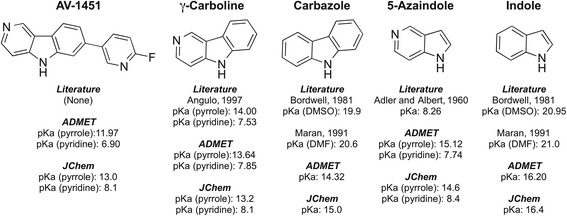
Predicted pKa of [^18^F]AV1451 by property prediction and comparison of analogues by literature and predicted pKa values indicates the gamma-carboline NH of [^18^F]AV1451 is more acidic than a typical indole, carbazole or azaindole

**Scheme 2:**
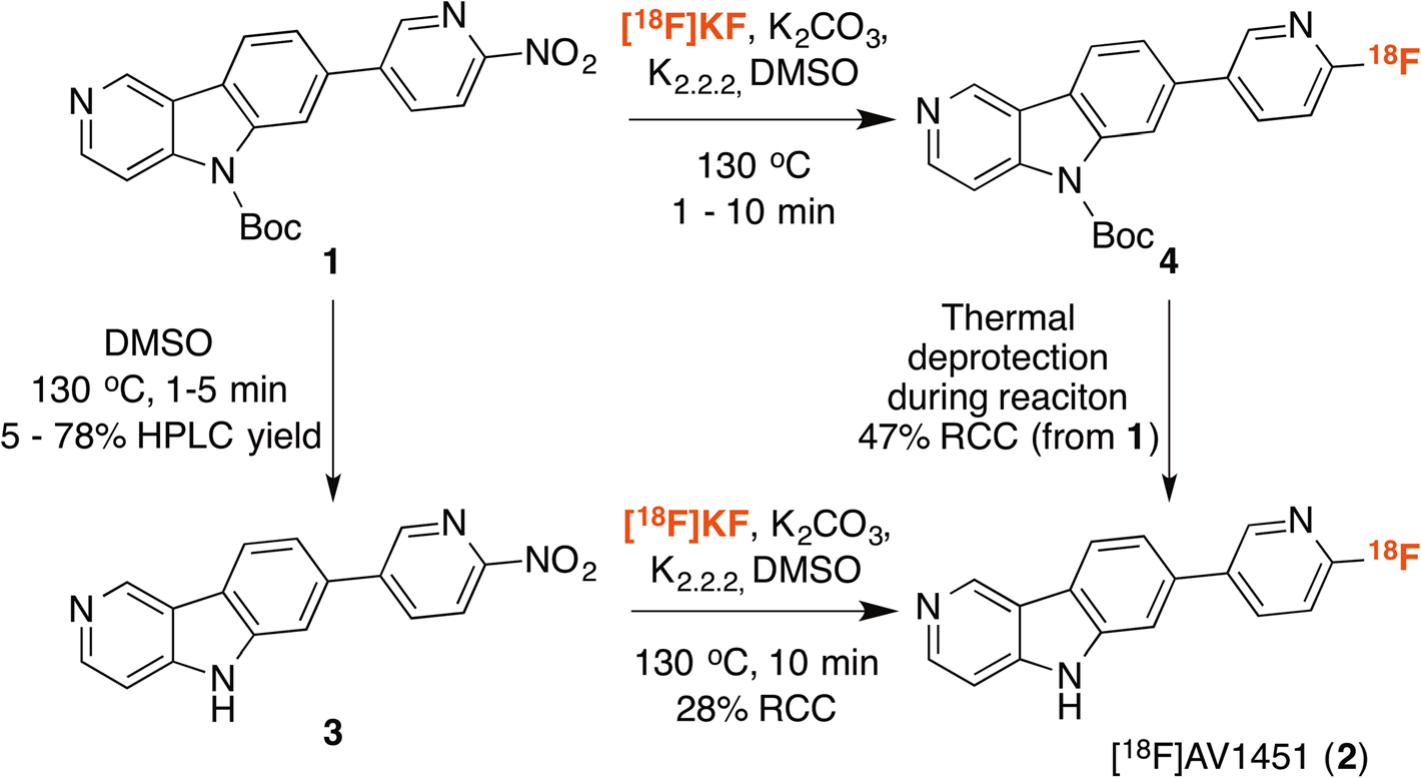
Investigating the synthesis of [^18^F]AV1451

**Fig. 5:**
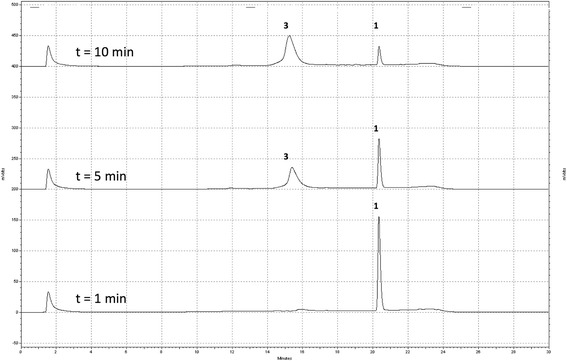
HPLC traces at 1 min, 5 min and 10 min of AV1451 precursor in DMSO at 130 °C

To evaluate the feasibility of a thermal deprotection we evaluated the predicted pKa of AV1451 versus predicted and literature values of analogues to determine why AV1451 would behave differently than a typical carbazole or indole (pKa values sourced either from the literature and/or predicted with JChem (https://epoch.uky.edu/ace/public/pKa.jsp) or ADMET Predictor 7.2 (courtesy of Adam C. Lee, PhD at Dupont) are summarized in Fig. 4). We found that γ-carbolines (Angulo et al. 1997) are more acidic than corresponding carbazole (Bordwell et al. 1981; Maran et al. 1991), azaindole (Adler and Albert 1960) and indole (Bordwell et al. 1981; Maran et al. 1991) and reasoned this observed change in electronics could explain why thermal removal of an N-Boc protecting group is possible with [^18^F]AV1451. To experimentally determine the extent of thermal deprotection, reactions were run (Scheme 2) and analyzed by HPLC (Fig. 5). N-Boc nitro precursor **1** was dissolved in DMSO and heated at 130 °C. Aliquots were removed at 1, 5 and 10 minutes and analyzed by HPLC. Within 1 min, the ratio of N-Boc precursor **1** (t_R_ = 20.3 min) : deprotected precursor **3** (t_R_ = 14.7 minutes) was 95 : 5 (Fig. 5). Greater conversion was observed at 5 min (**1** : **3** = 52 : 48), and at 10 min the majority of N-Boc precursor **1** was thermally deprotected (**1** : **3** = 22 : 78). These results confirmed the feasibility of thermal deprotection, and that basic or acidic conditions are not required for the removal of the N-Boc protecting group during the synthesis of [^18^F]AV1451.

**Fig. 6:**
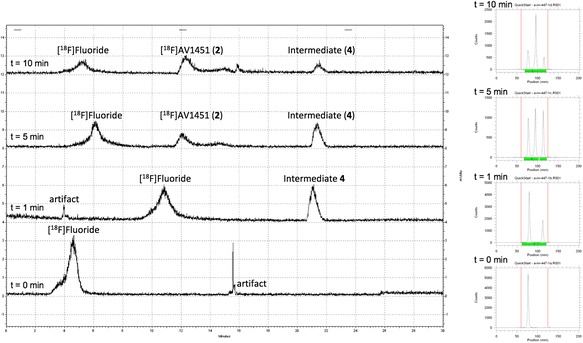
Radio-TLC (right) and -HPLC (left) data from manual [^18^F]AV1451 radiolabeling reactions, showing formation of the [^18^F]-N-Boc-protected intermediate **4**, followed by the [^18^F]AV1451 product **2**. Aliquots were pulled at t = 0, 1, 5, and 10 minutes and analyzed using both methods [Radio-TLC analysis was performed using a Bioscan AR 2000 Radio-TLC scanner with EMD Millipore TLC silica gel 60 plates (3.0 cm wide x 6.5 cm long) developed using hexane:ethyl acetate (1:1); Radio-HPLC analysis was performed using a Shimadzu LC2010 HPLC equipped with radioactivity and UV detectors (column: Luna C18 5 micron 4.6x150 mm; MeCN:H_2_O gradient: 5:95 (0–3 min) – 5:95 → 95:5 (3–20 min) – 95:5 → 5:95 (20–30 min); flow rate: 1.5 mL/min; UV: 254 nm). Note: [^18^F]fluoride t_R_ tended to fluctuate with the unbuffered H_2_O/MeCN HPLC gradient, and is thus present at different times in the HPLC rad traces (4–11 min), while small peaks observable in HPLC traces of t = 0 and 1 minutes are artifacts]

To study the radiofluorination reaction, [^18^F]KF/K_2_CO_3_/K_2.2.2_ was prepared as a stock solution in DMSO and reactions with the N-Boc-protected nitro precursor were carried out manually. Two products were observed by radio-TLC and radio-HPLC (Scheme 2 and Fig. 6), corresponding to the [^18^F]N-Boc intermediate **4** (t_R_ = 21–22 min) and [^18^F]AV1451 **2** (t_R_ = 12 – 13 min). Aliquots taken at 1 min demonstrated that the intermediate was the major radiolabeled product and that the precursor was still N-Boc protected. The RCC of the intermediate at this time was 43.4% with only 5.5% RCC to [^18^F]AV1451. At 5 min the RCC to intermediate **4** and [^18^F]AV1451 **2** were 47% and 23%, respectively. Finally, after a 10 min reaction at 130 °C, thermal deprotection of the Boc group resulted in 47% RCC to [^18^F]AV1451 (23% of the activity balance remained as intermediate **4**). Interestingly, we noted that conversion of [^18^F]KF to intermediate **4** (or product **2**) did not appreciably increase from 5 to 10 min.

We were uncertain if the lack of radiochemical conversion to intermediate or product from 5 to 10 min was due to the deprotection of the precursor (only 40% of N-Boc precursor remained at 5 min) or another reason. Therefore, N-Boc precursor **1** was first thermally deprotected to give **3**, and **3** was then subjected to fluorination. Radiolabeling was successful (Scheme 2), but only generating [^18^F]AV1451 **2** in a modest 28% RCC, suggesting that the non-protected precursor **3** can be radiolabeled so long as it is solubilized. This also suggests that the modest RCC observed in the radiofluorination of **1**, and decline in further reaction after 5 min, is likely due to the decreased solubility (and thus lower concentration) of precursor associated with thermal deprotection of the Boc group as the reaction proceeds. The results also indicate that the benefit of N-Boc protection in the reaction is primarily the improvement of precursor solubility.

The methods reported by Shoup and Gao dealt with the issue of purifying [^18^F]AV1451 **2** from nitro precursor **1** (and other reaction impurities such as **3** and **4**) in different ways. Shoup addressed the issue by adding a SPE extraction prior to HPLC purification. The reaction mixture was diluted in water and passed through an HLB cartridge, which removed DMSO and hydrophilic impurities, while trapping the product and other lipophilic components. The HLB cartridge was eluted with ethanol into the intermediate vial, which contained a low pH HPLC buffer. The removal of DMSO and hydrophilic impurities that would otherwise complicate subsequent semi-preparative HPLC purification allowed them to achieve separation. However, the pre-purification process requires the commercial system to be reconfigured as extra valves need to be added (Shoup et al. 2013). This increases operational complexity and also the number of steps (synthesis time). On the other hand, Gao reported two different methods to purify [^18^F]AV1451 (Gao et al. 2015). The first method involved using Fe(0)/formic acid to reduce the 2-nitropyridine to the 2-aminopyridine following radiolabeling, similar to the approach taken in the original synthesis (Xia et al. 2013). This requires extensive modification of a commercial synthesis system, and also increases potential points of failure in the synthesis. The second method described by Gao is more interesting: the reaction is quenched by addition of a 0.1 M NaHCO_3_ before purification with an acidic HPLC buffer (25% MeCN, 20 mM H_3_PO_4_). While there are not sufficient details in the report to know the extent of separation, we were intrigued that a simple basic quench with bicarbonate solution was sufficient to allow purification of [^18^F]AV1451.

We reasoned from the calculated pKa of [^18^F]AV1451 (Fig. 4), as well as a literature report of the pKa of γ-carboline (Angulo et al. 1997), that the use of a basic medium was the optimal approach to purify this radiotracer. The γ-carboline core of [^18^F]AV1451 contains both pyrrole-like and pyridine-like nitrogen atoms. ADMET and JChem predict the pKa of the pyrrole NH is ~12-13, and the conjugate pKa of the pyridine is ~7-8 (Fig. 4), and so the best separation could likely be achieved at a pH where [^18^F]AV1451 (and other reaction components) would be neutral. Since we also wished to avoid the use of Class 2 solvents such as MeCN, we elected to use an ethanolic phosphate buffer at pH 9.3. This buffer will leave the pyrrole nitrogen protonated, but will not protonate the pyridine-like nitrogen of the γ-carboline core. A comparison of the reported acidic buffers with this basic buffer revealed sharper peaks and increased separation when using the basic buffer (data not shown). Moreover, peak broadening and overlap of [^18^F]AV1451 **2** with contaminants, that occurred when we used acidic HPLC mobile phases, was not an issue with the basic buffer. The longer retention times and better peak shapes gave baseline separation of the product from other reaction components.

Finally, with a better appreciation of the synthesis, deprotection and purification of [^18^F]AV1451, we wished to automate the production of [^18^F]AV1451 (Scheme 1) using a TRACERLab FX_FN_ synthesis module. We initially re-optimized radiolabeling and purification using a smaller amount of precursor (0.5 mg vs 1.5 mg) and solvent (0.5 mL vs 1.5 mL), in order to further reduce HPLC peak broadening and also avoid precipitation prior to injection onto the HPLC column. Following the concomitant radiofluorination/Boc deprotection, the reaction mixture was purified by semi-preparative HPLC (column: Gemini NX C18 5 micron 10x250 mm; mobile phase: 40% Ethanol 10 mM Na_2_HPO_4_ pH: 9.3 ± 0.2; flow rate: 3 mL/min; for a typical semi-preparative HPLC trace, see Fig. 1 ([^18^F]AV1451 **2** t_R_ = 21–22 min)). This semi-preparative HPLC system showed a good retention time difference between the product and other reaction components that allowed for routine clinical production. A subsequent reformulation using an Oasis HLB cartridge provided [^18^F]AV1451 (**2**). Total synthesis time was 70 min and the method was validated for the manufacture of doses for clinical use by completing four process-verification runs. The isolated, formulated and non-corrected radiochemical yield (RCY) of [^18^F]AV1451 prepared using this method was 202 ± 57 mCi at end-of-synthesis (EOS) (14% based on starting [^18^F]fluoride), RCP = 98 ± 1%, and SA = 2521 ± 623 Ci/mmol. Doses met (or exceeded) the QC specifications as described in the methods section (Table 1).

## Conclusion

In summary, an optimized fully automated synthesis of [^18^F]AV1451 has been developed which uses an unmodified TRACERLab FX_FN_ synthesis module. The synthesis also employs no hazardous solvents, and provides radiotracer doses suitable for clinical use. Based on our results in the development of this method, we encourage others to utilize pKa values to help determine the optimal mobile phase to purify radiotracers given the increasing availability of diverse HPLC stationary phases that can be used in combination with mobile phases of varying acidity (or basicity). The use of multiple manual reactions performed using aliquots of a batch prepared reagent (as was done with [^18^F]KF/K_2.2.2_ in DMSO to prepare [^18^F]AV1451 in this study) is also a convenient way to quickly assess a reaction, and the role of reagents and reaction conditions.

## Abbreviations

AD: Alzheimer’s disease
Boc: *tert*-butyloxycarbonyl protecting group
CBD: Corticobasal degeneration
DMSO: Dimethyl sulfoxide
EOS: End-of-synthesis
K_2.2.2_: Kryptofix-2.2.2
MeCN: Acetonitrile
PET: Positron emission tomography
PSP: Progressive supranuclear palsy
QC: Quality control
QMA: Quaternary methyl ammonium
RCC: Radiochemical conversion
RCP: Radiochemical purity
RCY: Radiochemical yield
SA: Specific activity
SPE: Solid-phase extraction
t_R_: Retention time
